# Biochemical characterization of a novel acidophilic β-xylanase from *Trichoderma asperellum* ND-1 and its synergistic hydrolysis of beechwood xylan

**DOI:** 10.3389/fmicb.2022.998160

**Published:** 2022-09-15

**Authors:** Fengzhen Zheng, Abdul Basit, Huan Zhuang, Jun Chen, Jianfen Zhang, Weiqing Chen

**Affiliations:** ^1^College of Biological and Environmental Engineering, Zhejiang Shuren University, Hangzhou, China; ^2^Department of Microbiology, University of Jhang, Jhang, Pakistan; ^3^Department of ENT and Head & Neck Surgery, The Children’s Hospital Zhejiang University School of Medicine, Zhejiang, Hangzhou, China; ^4^Interdisciplinary Research Academy, Zhejiang Shuren University, Hangzhou, China

**Keywords:** *Trichoderma asperellum*, β-xylanase, acidophilic, mode of action, synergism

## Abstract

Acidophilic β-xylanases have attracted considerable attention due to their excellent activity under extreme acidic environments and potential industrial utilizations. In this study, a novel β-xylanase gene (*Xyl11*) of glycoside hydrolase family 11, was cloned from *Trichoderma asperellum* ND-1 and efficiently expressed in *Pichia pastoris* (a 2.0-fold increase). Xyl11 displayed a maximum activity of 121.99 U/ml at pH 3.0 and 50°C, and exhibited strict substrate specificity toward beechwood xylan (*K*_m_ = 9.06 mg/ml, *V*_max_ = 608.65 μmol/min/mg). The Xyl11 retained over 80% activity at pH 2.0–5.0 after pretreatment at 4°C for 1 h. Analysis of the hydrolytic pattern revealed that Xyl11 could rapidly convert xylan to xylobiose via hydrolysis activity as well as transglycosylation. Moreover, the results of site-directed mutagenesis suggested that the Xyl11 residues, Glu^127^, Glu^164^, and Glu^216^, are essential catalytic sites, with Asp^138^ having an auxiliary function. Additionally, a high degree of synergy (15.02) was observed when Xyl11 was used in association with commercial β-xylosidase. This study provided a novel acidophilic β-xylanase that exhibits excellent characteristics and can, therefore, be considered a suitable candidate for extensive applications, especially in food and animal feed industries.

## Introduction

Hemicellulose, the second most abundant carbon source in crop wastes, is composed of lignin and cellulose and thus constitutes the lignocellulose of plants (Davidi et al., 2016). Xylan is the major hemicellulosic ingredient and can be converted into highly valuable chemicals and industrial products (Ragauskas et al., 2006; Aachary and Prapulla, 2008). It consists of a backbone of β-1,4-linked xylopyranose monomer and side chains of diverse substituents (Basit et al., 2018a). Biocatalysts from natural sources like microorganisms have gained much attention due to many potential applications like bioenergy, biofuel production, biobleaching, bioconversion and so on (Golgeri et al., 2022). Efficient hydrolysis of xylan can be achieved using the action of various efficient enzymes that transform hemicellulosic biomass into valuable soluble sugars (Banerjee et al., 2010; Bouchat et al., 2022). Xylanases are a major subgroup of hemicellulases that catalyze xylan degradation and possess extensive potential applications, especially in animal feed and food fields (Juturu and Wu, 2012; Bagewadi et al., 2016; Basit et al., 2019).

Enzymatic hydrolysis for xylan deconstruction is generally inefficient, mainly due to the high cost of enzymes production (Huang et al., 2015; Taylor et al., 2018). Genetic engineering techniques have facilitated large-scale production of recombinant proteins using *Pichia pastoris* (Basit et al., 2018b). It generates relatively few native proteins and heterologous proteins can be secreted into culture media through directing signal peptides (Karbalaei et al., 2020). Protein expression levels have been considerably improved by regulating several factors, including directed evolution and codon optimization (Yang and Zhang, 2018). Hence, high-efficiency expression of xylanase in *P. pastoris* is highly recommended for its low cost.

Production of xylose is generally depends on synergistic actions of multiple glycoside hydrolases (GHs), particularly β-xylanases, and β-xylosidases (Yang et al., 2014; Zhuo et al., 2018). Among them, β-xylanase is the key enzyme as it initially degrades the xylan backbone to liberate xylooligosaccharides (XOSs) with a degree of polymerization (DP) ranging from 2 to 10 units (Santibáñez et al., 2021); these XOSs are further converted into xylose by β-xylosidase. Currently, β-xylanases are categorized into different GH groups, including GH51, GH43, GH30, GH11, GH10, and GH8 (Collins et al., 2005). Furthermore, β-xylanases of GH11 family are generally applied in biomass conversion and food processes due to their low molecular weight (Mw) and high degradation efficiency (Juturu and Wu, 2012; Falck et al., 2013).

β-xylanases from different GH families and sources display great diversity in hydrolytic properties (Valenzuela et al., 2010; Moreira and Filho, 2016). In particular, β-xylanases that are acidophilic and acid-stable have preferable application prospects in various fields, since acidic environments can decrease the risk of microbial contamination (Juturu and Wu, 2012; Yu et al., 2021). Recently, a great deal of acidophilic β-xylanases have been identified in fungi (Luo et al., 2009; Teng et al., 2019). For example, the β-xylanases of *Penicillium oxalicum* GZ-2 (Liao et al., 2014) and *Thermoascus aurantiacus* M-2 (Ping et al., 2018), belonging to the GH11 family, showed their highest activity at pH 4.0 and 5.0, respectively. The GH10 β-xylanases secreted by fungi, such as *Phialophora* sp. G5 (Zhang et al., 2011), *Scytalidium candidum* 3C (Eneyskaya et al., 2020), and *Aureobasidium pullulans* var. *Melanigenum* ATCC 20524 (Ohta et al., 2001), displayed the highest activity at pH 4.0, 3.5, and 2.0, respectively. These enzymes under such special conditions are important for converting biomass into value-added products. Among the diverse microorganisms that yield lignocellulolytic enzymes, *Trichoderma asperellum* is known for its significant biomass hydrolysis ability (Marx et al., 2013). Compared with *T. reesei* QM6a, the genome of *T. asperellum* ND-1 encoded a unique enzymatic system, especially xylanolytic enzymes (Zheng et al., 2022). However, the action model and synergistic effect of acidophilic β-xylanases from *T. asperellum* have rarely been reported.

In this study, a novel acidophilic β-xylanase gene (*xyl11*) from *T. asperellum* ND-1 was cloned and efficiently expressed in *P. pastoris* through codon optimization. Essential catalytic sites responsible for the β-xylanase (Xyl11) activity were analyzed by site-directed mutagenesis, the mode of action on XOSs was investigated, and the synergistic effect of Xyl11 in combination with commercial β-xylosidase on the hydrolysis of beechwood xylan was evaluated.

## Materials and methods

### Materials

*Trichoderma asperellum* ND-1 (GenBank no: MH496612) was kept in our laboratory. *P. pastoris* X-33, *Escherichia coli* DH5α, and pPICZαA plasmid were procured from Invitrogen (CA, United States). *p*-Nitrophenyl (pNP)-α-L-arabinofuranoside (pNPAf), *p*-nitrophenyl-β-D-cellobioside (pNPC), *p*-nitrophenyl-β-D-galactopyranoside (pNPG), and sodium carboxymethyl cellulose (CMC-Na) were procured from Sigma-Aldrich (United States). Xylopentaose (X5), xylotetraose (X4), xylotriose (X3), xylobiose (X2), xylose (X1), beechwood xylan and β-xylosidase (EC 3.2.1.37, *Selenomonas ruminantium*) were purchased from Megazyme (Wicklow, Ireland). Corncob xylan (CXY; 30% X2, 40% X3, 15% X4, and 15% X5) was procured from Shanghai Aladdin Biochemical Technology (Shanghai, China). Restriction endonucleases (*Xba*I and *Eco*RI) were obtained from TaKaRa (Tokyo, Japan). Mut Express II Fast Mutagenesis Kit V2 was procured from Vazyme Biotech Co., Ltd., (Nanjing, China).

### Cloning of β-xylanase gene (*xyl11*) from *Trichoderma asperellum* ND-1

The whole *xyl11* gene of *T. asperellum* ND-1 was cloned from its genome sequence (Gene ID: OM128443, 693 bp). Signal peptides were analyzed using SignalP-5.0 server.^1^ Using the cDNA of *T. asperellum* ND-1 as template, a pair of primers Xyl11-F and Xyl11-R (Supplementary Table 1), was used to amplify the *xyl11* gene without a signal peptide-coding sequence. The optimization of *xyl11* gene was achieved based on the preferred codon usage of *P. pastoris* and designated as *oxyl11*.^2^

### Construction of high-level expression strains

**FIGURE 1:**
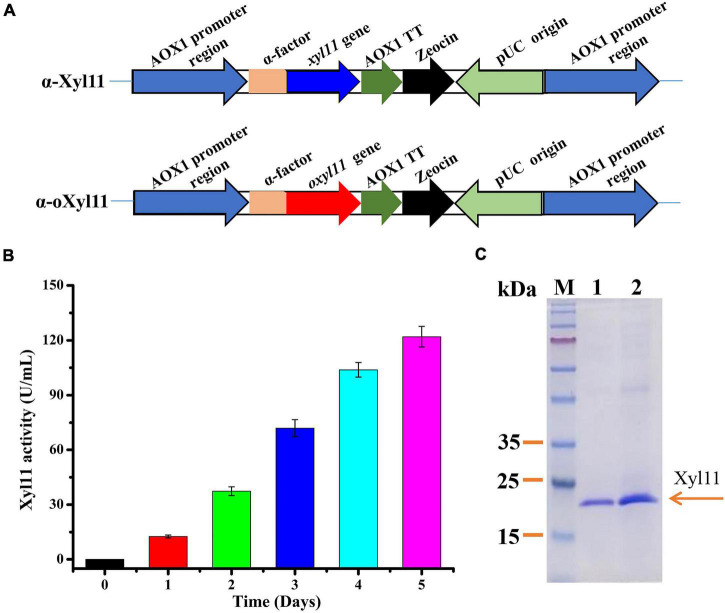
High-efficiency expression of Xyl11 in *P. pastoris*. **(A)** Schematic representation of recombinant strains α-Xyl11 (containing original *xyl11* gene) and α-oXyl11 (containing optimized *oxyl11* gene). **(B)** Extracellular enzyme activity of recombinant strain α-oXyl11. **(C)** SDS-PAGE analysis of proteins expressed by α-Xyl11 and α-oXyl11. Lane M: standard protein markers; Lane 1: fermentation supernatant of α-Xyl11 in shake flasks; Lane 2: fermentation supernatant of α-oXyl11 in shake flasks.

The PCR product was extracted from the gel using a Gel Extraction Kit (TaKaRa, Japan) and connected to the pPICZαA expression vector pre-digested with *Eco*RI and *Xba*I. The constructed vectors were converted into *E. coli* DH5α and verified using PCR. The constructed vectors containing *xyl11* and *oxyl11* were named pPICZα-*xyl11* and pPICZα-*oxyl11*, respectively. The recombinant vectors were both linearized using *Sac*I and electroporated into *P. pastoris* competent cells to obtain the engineered strains, α-Xyl11 and α-oXyl11, as shown in Figure 1A. The recombinants were selected on peptone dextrose medium (YPD) agar plates containing 100 μg/ml Zeocin (Invitrogen, United States) (Basit et al., 2019). The positive transformants were further confirmed by PCR using the AOX-F/AOX-R primer pair.

### Confirmation of copy number of *xyl11* gene in engineered strain *Pichia pastoris*

The copy number of the *xyl11* gene in *P. pastoris* was identified by PCR using a specific primer (pUC-F/*xyl11* internal reverse primer*-*R) (Supplementary Table 1); the schematic diagram is presented in Supplementary Figure 1A. Genomes of the wild strain X-33 (without target gene), and the engineered strains (α-Xyl11 and α-oXyl11) were obtained as reported previously (Kumar and Satyanarayana, 2015).

### Protein expression and SDS-PAGE

The positive single colonies of α-Xyl11, α-oXyl11, and X-33 were incubated in 5 ml YPD at 30°C while shaking at 200 rpm for 12 h. The expression of transformants in buffered methanol-complex (BMMY) was analyzed as reported previously (Zheng et al., 2018). Culture supernatants were examined using 12% SDS-PAGE, stained with Coomassie brilliant blue R-250 (Bio-Rad Laboratories, United States), and de-stained with 100 ml/L ethyl alcohol and 100 ml/L acetic acid. The concentrations of protein were evaluated using the Bradford method (Bradford, 1976).

### Biochemical characterization of Xyl11

Xyl11 activity was tested with the 3,5-dinitrosalicylic acid (DNS) method using X1 as the standard (Miller, 1959). The reaction contents, including 100 μl of 1% (w/v) beechwood xylan and 100 μl of diluted crude enzyme, were incubated in 50 mM glycine-HCl buffer (pH 3.0) for 10 min at 50°C, and then stopped with 50 μl of 1 M NaOH. The quantity of reducing sugar was evaluated at 540 nm.

Effects of pH on Xyl11 activity were measured in the pH range 2.0–8.0, using the different buffers (each 50 mM): glycine-HCl (pH 2.0–3.0), sodium citrate (pH 3.0–4.0), sodium acetate (pH 4.0–6.0), and sodium phosphate (pH 6.0–8.0). Mixtures without the enzyme were defined as controls. Effects of pH on enzyme stability were tested by determining the residual activity following pretreatment in each buffer for 1 h at 4°C without substrate. To determine its half-lives at pH 2.0–5.0, Xyl11 was pre-incubated at 4°C for various times in the above buffers without substrate.

The optimal temperature for Xyl11 was analyzed using 50 mM glycine-HCl buffer (expressed as relative activity) in the range of 20–80°C. Thermostability of Xyl11 was examined by calculating the residual activity following pretreatment at different temperatures for 30 min.

The effects of various metal ions and chemical reagents on enzyme activity were evaluated by adding 1 or 5 mM of the respective metal ion (Mg^2+^, NH_4_^+^, Li^+^, Ni^2+^, Fe^3+^, Fe^2+^, Na^+^, K^+^, Cd^2+^, Zn^2+^, Al^3+^, Ba^2+^, Ca^2+^, Co^2+^, Pb^2+^, Mn^2+^, or Cu^2+^), and chemical reagents (SDS, urea, and EDTA). Xyl11 was pretreated in 50 mM glycine-HCl buffer (pH 3.0) for 60 min in the presence of 1 or 5 mM of various reagents at 4°C. Mixtures without additives were defined as controls. The residual activity was then evaluated using the standard method. All experiments were analyzed in triplicate.

### Substrate specificity and kinetic parameters

To estimate the substrate specificity of Xyl11, its activity was assessed using pNPG, pNPC, pNPAf, and pNPX as substrates. The reaction mixture [100 μl Xyl11 in 50 mM glycine-HCl buffer (pH 3.0), with 100 μl substrate (5 mM)] was treated for 10 min at 50°C, and the reaction was stopped by adding 100 μl sodium carbonate (1.0 M). The amount of pNP released was assayed by measuring at 405 nm, and one activity unit was defined as the amount of enzyme that released 1 μmol pNP per minute. For wheat arabinoxylan, CMC-Na, and beechwood xylan, the enzyme activity was determined using the DNS method as described above.

The kinetic parameters, *V*_max_ and *K*_m_ for Xyl11 were calculated using the Lineweaver-Burk plot method (Erithacus Software, Horley, United Kingdom). Enzyme activities were measured using 1–10 mg/ml of beechwood xylan as the substrate in glycine-HCl buffer (pH 3.0) at 50°C. The experiments were performed in triplicate.

### Hydrolytic properties of Xyl11

To estimate the hydrolytic properties of Xyl11, the hydrolysis products from CXY and XOS standards (X1-X5) were analyzed. A total of 10 U/ml Xyl11 was incubated with 10 mg/ml CXY or 5 mg/ml XOS standards at 50°C for 2 h in glycine-HCl buffer (pH 3.0). Substrates containing inactive enzyme were used as blank controls. All the samples were boiled for 5 min after incubation and centrifuged at 12,000 rpm and 4°C for 15 min. The supernatants were further analyzed using an LC-20A HPLC system (Shimadzu, Japan) equipped with an ROA-Organic Acid H^+^ (8%) column (Phenomenex) and an RIDL10A refractive index detector, and a column temperature of 50°C, and 5 mM H_2_SO_4_ at a flow rate of 0.6 ml/min. X1-X5 standards were purchased from Megazyme.

### Construction of Xyl11 mutants

The catalytic sites of Xyl11 were considered to be Glu^127^ and Glu^216^ based on the BLAST information.^3^ SWISS-MODEL software was employed to simulate the 3D structure of Xyl11, with endo-1,4-xylanase from *Trichoderma reesei* (PDB ID: 1xyn.1) as the template (sequence similarity 65.65%) (Törrönen and Rouvinen, 1995).^4^ Based on the template structure and multiple sequence alignment, residues Asp^58^, Asp^84^, Asp^138^, Asp^204^, and Glu^164^ were also responsible for Xyl11 activity. To verify the critical catalytic residues, Xyl11 mutants named D58A, D84A, D138A, D204A, E127A, E164A, and E216A were generated via site-directed mutagenesis corresponding to sites Asp^58^, Asp^84^, Asp^138^, Asp^204^, Glu^127^, Glu^164^, and Glu^216^, respectively. Recombinant plasmids containing the mutant genes were cloned from the constructed pPICZα-*oxyl11* plasmid with the specified primers (Supplementary Table 1) using Phanta Max Super-Fidelity DNA Polymerase one-step PCR. The resulting mutant plasmids were converted into *P. pastoris*. Protein expression and activity determination were carried out as described above. *Pichia pastoris* containing the normal gene (*oxyl*) served as the control.

### Synergistic effect of Xyl11 and commercial β-xylosidase

The enzymatic degradation of 10 mg/ml beechwood xylan by Xyl11 (15 U/ml) and β-xylosidase (25 U/ml) individually or in combination was performed in glycine-HCl buffer (pH 3.0) at 50°C. Samples were collected after different time intervals for enzyme activity evaluation. The reaction of each collected sample was terminated by boiling for 10 min and centrifuged at 12,000 rpm, 4°C for 15 min to collect the supernatant. The hydrolysates were assayed using HPLC, as described above. The experiments were carried out in triplicate.

## Results and discussion

### Sequence analysis of *xyl11* gene

The full-length β-xylanase gene (*xyl11*) from *T. asperellum* ND-1 was 693 bp, encoding 230 amino acids; the sequence has been submitted to the NCBI database under accession number OM128443. According to the CAZymes database, the novel β-xylanase (termed Xyl11) falls in the GH11 family.^5^ After codon optimization, the GC content of the *xyl11* gene increased from 53.8 to 58.3% (Supplementary Figure 2) and the codon adaptation index improved from 0.68 to 0.91. SignalP-5.0 server analysis revealed that Xyl11 had a 19-amino-acid (MVAFSSLFVAFAGFTGVLA) signal peptide and the theoretical Mw of the mature protein was 22,475 Da, which was calculated using DNAMAN6.0 software.

### Expression of Xyl11 in *Pichia pastoris*

*Pichia pastoris* is a widely used host for recombinant protein expression. According to the literature, various methods can be utilized to achieve high-level expression of heterologous proteins (Yang and Zhang, 2018). Different microorganisms have various preferred codons, and codon optimization of the target gene expressed in *P. pastoris* is a desirable and effective strategy (Looser et al., 2015). Hence, the impact of optimization of *xyl11* codons on enzyme activity was analyzed. Nucleotide sequence alignments showed that the optimized gene (*oxyl11*) shared 74.89% identity with the original gene (*xyl11*), with 173 nucleotides being modified (Supplementary Figure 2). The engineered strains, α-oXyl11 and α-Xyl11, were constructed (Figure 1A), and confirmed using PCR (Supplementary Figures 1B,C). All strains contained only one copy of the *xyl11* gene (Supplementary Figure 1D). Wild-type strain X-33 was utilized as blank control.

After methanol induction in BMMY medium, the Xyl11 activity of the α-oXyl11 engineered strain continuously increased, reaching a maximum of 121.99 ± 5.66 U/mL (Figure 1B), which was about 2.0-fold higher than that of the original strain, α-Xyl11 (65.25 ± 2.86 U/ml). Moreover, Xyl11 specific activity of recombinant strain α-oXyl11 (393.52 ± 18.25 U/mg) was significantly higher than xylanases from *Penicillium oxalicum* GZ-2 (150.2 U/mg) (Liao et al., 2014), *Bacillus amyloliquefaciens* (128.75 ± 1.5 U/mg) (Liu et al., 2017), and *Penicillium sclerotiorum* (249.15 U/mg and 240.89 U/mg) (Knob and Carmona, 2010). It has been reported that codon optimization can efficiently increase the expression levels of heterologous proteins in *P. pastoris*, such as the β-xylanase of *Aspergillus sulphureus* JCM01963 (Liu et al., 2021), α-L-arabinofuranosidase of *Aspergillus niger* ND-1 (Zheng et al., 2018), and β-xylanase of *Chaetomium* sp. CQ31 (Yu et al., 2021). However, codon optimization has hitherto not been utilized to increase the yields of acidophilic β-xylanases from *T. asperellum*. The results of this study indicated that codon optimization could remarkably influence the biological activity of the acidophilic β-xylanase of *T. asperellum* ND-1 in *P. pastoris*.

As shown in Figure 1C, comparative analysis of the fermentation supernatant of α-oXyl11 and α-Xyl11 by SDS-PAGE revealed that the Mw of Xyl11 was 22 kDa, which was almost identical to the theoretical Mw of 22,475 Da. It has been reported that numerous GH11 β-xylanases display Mw values within the range of 18 to 30 kDa (Paës et al., 2012). Moreover, the target protein (Xyl11) accounted for the major portion of the secreted proteins in the fermentation supernatant (Figure 1C), making it significantly cost-effective for various industrial applications.

### Enzymatic characterization of Xyl11

**FIGURE 2:**
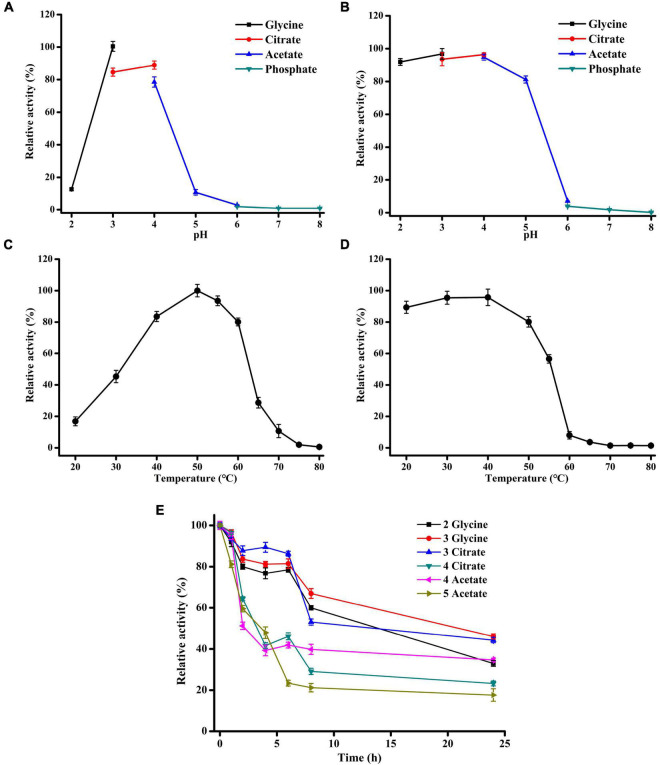
Effects of pH and temperature on Xyl11 activity. **(A)** Optimal pH. The enzyme activity was tested at 50°C in pH range 2.0–8.0, using different buffers (each 50 mM). **(B)** pH stability of Xyl11 was tested after treatment in different buffers at 4°C for 1 h. **(C)** Optimal temperature of Xyl11 was analyzed using glycine-HCl buffer (pH 3.0) within the range 20–80°C. **(D)** Thermostability of Xyl11 was evaluated after treatment within the range 20–80°C for 30 min. **(E)** The half-lives of Xyl11 at pH 2.0–5.0. Residual activities were assayed at pH 2.0–5.0 after incubation of the enzyme for different periods of time. All experiments were carried out in triplicate.

The effects of temperature and pH on the activity of Xyl11 produced by α-oXyl11 were evaluated using 10 mg/ml beechwood xylan as the substrate. Xyl11 displayed the maximum activity at pH 3.0 in glycine-HCl buffer (Figure 2A). Similar pH optima of β-xylanase have been reported for *Penicillium oxalicum* GZ-2 (Liao et al., 2014), *P. sclerotiorum* (Knob and Carmona, 2010), *Bispora* sp. MEY-1 (Luo et al., 2009), *Penicillium citrinum* HZN13 (Bagewadi et al., 2016) and *A. pullulans* NRRL Y-2311-1 (Yegin, 2017). Moreover, Xyl11 exhibited significant tolerance to acidic pH, retaining over 80% of its original activity at pH 2.0–5.0 after pretreatment at 4°C for 1 h (Figure 2B). Besides, the half-lives of Xyl11 at pH 2.0–3.0 and pH 4.0–5.0 were 24 and 4 h, respectively (Figure 2E). The enzyme remained stable up to 6 h with above 80% activity at pH 3.0, and retained 32% activity at pH 2.0 after 48 h (Figure 2E). The stability at acidic pH indicates the potential for the utilization of Xyl11 in different industrial fields such as animal feed, food, and bioenergy fields (Knob and Carmona, 2010). In biocatalytic sectors, acidic pretreatments are generally performed prior to or simultaneously with enzymatic hydrolysis; hence, utilizing the acidophilic and acidic-stable Xyl11 in low pH environments would be attractive (Juturu and Wu, 2012; Liao et al., 2014).

The temperature optima of Xyl11 was examined at 50°C, and the enzyme retained > 80% of the highest activity in the temperature range of 40–60°C (Figure 2C). However, temperature above 60°C caused a rapid reduction in Xyl11 activity (Figure 2C). Fungal β-xylanases generally have optimum temperature between 40 and 65°C (Carvalho et al., 2017). The thermostability of Xyl11 was also investigated (Figure 2D). More than 80% of the original activity was maintained after 30 min incubation at 20–50°C (Figure 2D). When incubated at 55°C for 30 min, 56.6% of the activity was retained (Figure 2D). However, the enzyme lost activity rapidly and completely after incubation at above 60°C for 30 min (Figure 2D). The thermal stability of Xyl11 may also make it a good candidate for feed additive under intestinal temperature.

The effects of various chemicals and metal ions on Xyl11 activity were investigated (Table 1). The presence of metal ions at 1 mM (Al^3+^, Mn^2+^, Cu^2+^, Co^2+^, Cd^2+^, and Pb^2+^) had partial or no influence on Xyl11 activity. The residue metal ions evaluated in this study displayed positive effect on Xyl11 activity (Table 1). Mg^2+^ (1 mM, 121.88%), Li^+^ (1 mM, 121.78%), K^+^ (1 mM, 121.34%), Na^+^ (1 mM, 125.77%), Zn^2+^ (1 mM, 123.61%), and Ba^2+^ (1 mM, 122.32%) remarkably stimulated the activity of Xyl11 (Table 1). Upon increasing the concentration to 5 mM, ions such as Ni^2+^, Mg^2+^, Fe^2+^, Cd^2+^, Al^3+^, Mn^2+^, Pb^2+^, Co^2+^, Ba^2+^, Cu^2+^, and Fe^3+^ appeared to inhibit enzyme activity; Pb^2+^ and Cu^2+^ strongly impaired Xyl11 activity by 46.32 and 37.14%, respectively (Table 1). This inhibitory action on Xyl11 activity may be because metal ions can bind to the active sites of xylanase, leading to structural changes (Ping et al., 2018). Furthermore, Xyl11 activity was evidently decreased in the presence of 5 mM EDTA (77.93%) and SDS (32.97%) (Table 1), as reported previously for xylanases from *Paenibacillus barengoltzii* (Liu et al., 2018), *Humicola insolens* Y1 (Du et al., 2013), *A. pullulans* NRRL Y-2311-1 (Yegin, 2017), and *Bacillus subtilis* Lucky9 (Chang et al., 2017). However, approximately 24% enhancement was induced by 1 mM urea, with SDS (101.63%) and EDTA (101.72%) having slight effects (Table 1).

**TABLE 1:** Effects of metal ions and chemical reagents on Xyl11 activity.

Chemicals	Relative activity (%)	
	1 mM	5 mM
Control	100.00 ± 3.44	100.00 ± 0.54
Mg^2+^	121.88 ± 1.26	81.51 ± 0.93
NH_4_^+^	113.01 ± 0.78	92.18 ± 0.60
Li^+^	121.78 ± 1.18	91.48 ± 0.42
Ni^2+^	117.30 ± 1.14	85.36 ± 0.18
Fe^2+^	118.43 ± 3.99	76.06 ± 0.68
K^+^	121.34 ± 2.40	98.17 ± 3.66
Na^+^	125.77 ± 2.91	92.60 ± 1.51
Cd^2+^	104.98 ± 1.23	74.54 ± 2.32
Zn^2+^	123.61 ± 1.82	92.99 ± 4.92
Al^3+^	89.95 ± 2.95	78.55 ± 3.46
Mn^2+^	90.29 ± 0.94	49.94 ± 0.87
Pb^2+^	90.54 ± 0.84	46.32 ± 3.37
Ca^2+^	117.00 ± 1.92	91.32 ± 2.19
Co^2+^	101.68 ± 2.36	70.53 ± 1.36
Ba^2+^	122.32 ± 2.19	89.41 ± 0.43
Cu^2+^	95.22 ± 1.80	37.14 ± 0.30
Fe^3+^	114.39 ± 3.54	49.09 ± 1.00
Urea	124.35 ± 1.90	95.56 ± 4.03
SDS	101.63 ± 0.37	32.97 ± 2.25
EDTA	101.72 ± 1.09	77.93 ± 1.74

### Substrate specificity and kinetic parameters of Xyl11

The Xyl11 secreted from α-oXyl11 was evaluated with different substrates to determine its specificity. Xyl11 displayed strict substrate specificity toward xylans. It showed maximal activity toward beechwood xylan (121.99 ± 5.66 U/ml), followed by wheat arabinoxylan (33.14 ± 3.02 U/ml). However, it had no activity toward CMC-Na, suggesting that Xyl11 is not efficient at cellulose degradation, similar to the endo-β-1,4-xylanases of *Penicillium citrinum* HZN13 (Bagewadi et al., 2016), *B. subtilis* Lucky9 (Chang et al., 2017), *P. barengoltzii* CAU904 (Liu et al., 2018), and *A. niger* NL-1 (Li et al., 2021). Cellulase-free β-xylanases can degrade hemicellulose selectively with minimal cellulose loss; they are hence beneficial for industrial applications, such as in the pulp bleaching and food industry (Belfaquih et al., 2002). Moreover, Xyl11 displayed no activity toward the degradation of pNP derivatives (PNPC, PNPG, and pNPAf).

The kinetics parameters of Xyl11 were calculated using beechwood xylan (1–10 mg/ml) under optimal conditions (50°C and pH 3.0). The *K*_m_ and *V*_max_ of Xyl11 were determined to be 9.06 mg/ml and 608.65 μmol/min/mg, respectively. The kinetic parameters of Xyl11 are consistent with the results exhibited by other fungal xylanases, ranging from 0.106 to 6300 μmol/min/mg for *V*_max_ and from 0.09 to 40.9 mg/ml for *K*_m_ (Beg et al., 2001). High *K*_m_ of xylanases have also been reported, such as for those from *A. pullulans* NRRL Y-2311-1 (19.43 mg/ml) (Yegin, 2017), *P. oxalicum* GZ-2 (30.7 mg/ml) (Liao et al., 2014), *Sorangium cellulosum* So9733-1 (25.77 mg/ml) (Wang et al., 2012), and *Penicillium citrinum* HZN13 (11.11 mg/ml) (Bagewadi et al., 2016). The lower *K*_m_ value revealed that Xyl11 showed greater affinity toward beechwood xylan compared to the aforementioned xylanases.

### Enzymatic cleavage pattern of Xyl11

**FIGURE 3:**
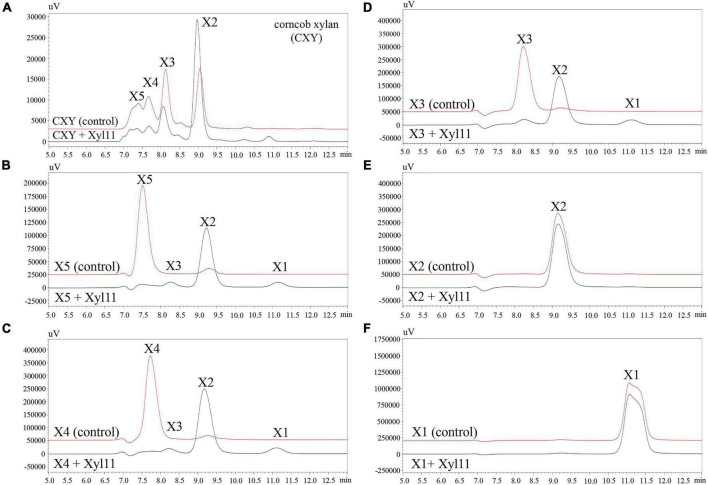
HPLC analysis of degradation products of corncob xylan **(A)**, XOS standards (DP 2–5) **(B–E)**, and xylose **(F)** by Xyl11. X5, xylopentaose; X4, xylotetraose; X3, xylotriose; X2, xylobiose; and X1, xylose. Control represents inactive Xyl11 treatment.

To investigate the hydrolysis patterns of Xyl11, the degradation products of CXY and XOSs (X1-X5) were investigated by HPLC. As shown in Figure 3, Xyl11 could efficiently degrade CXY to yield mainly X2; simultaneously, the proportions of X5, X4, and X3 decreased sharply (Figure 3A). Similarly, other xylanases from fungi such as *A. niger* BCC14405 (Aiewviriyasakul et al., 2021), *A. sulphureus* JCM01963 (Liu et al., 2021), and *Chaetomium* sp. CQ31 (Yu et al., 2021) hydrolyze xylans to yield predominantly X2. Moreover, X2 can be applied directly as a food additive and it displays remarkable prebiotic effects on intestinal probiotics, such as *Bifidobacterium* and *Lactobacillus* spp. (Rajagopalan et al., 2017; Santibáñez et al., 2021).

Further analysis revealed that the major degradation products of X5 and X4 were similar, mainly consisting of X2 with trace amounts of X3 and X1 (Figures 3B,C). Particularly, X3 was degraded into X2 without the formation of a correspondent amount of X1 (Figure 3D), suggesting that Xyl11 might have the function of transglycosylation. Several GH11 β-xylanases have also been reported to have a transglycosylation function, e.g., Xyn11E from *Paenibacillus barcinonensis* BP-23 (Valenzuela et al., 2014), XynST11 from *Streptomyces* sp. B6 (Liu et al., 2020), Tx-xyn11 from *Thermobacillus xylanilyticus* (Brusa et al., 2018), and β-xylanase from *Bacillus circulans* (Marneth et al., 2021). Additionally, Xyl11 was unable to degrade X2 into X1 (Figure 3E), and exhibited no activity toward X1 (Figure 3F), demonstrating that it was a typical endo-acting β-xylanase. It is well known that enzyme preparations with much lower β-xylosidase or exo-xylanase activity are attractive tools (Ho et al., 2018; Amorim et al., 2019); hence, Xyl11 is more suitable for X2-enriched XOS prebiotic production.

### Elucidation of hydrolysis patterns of Xyl11 via site-directed mutagenesis

**FIGURE 4:**
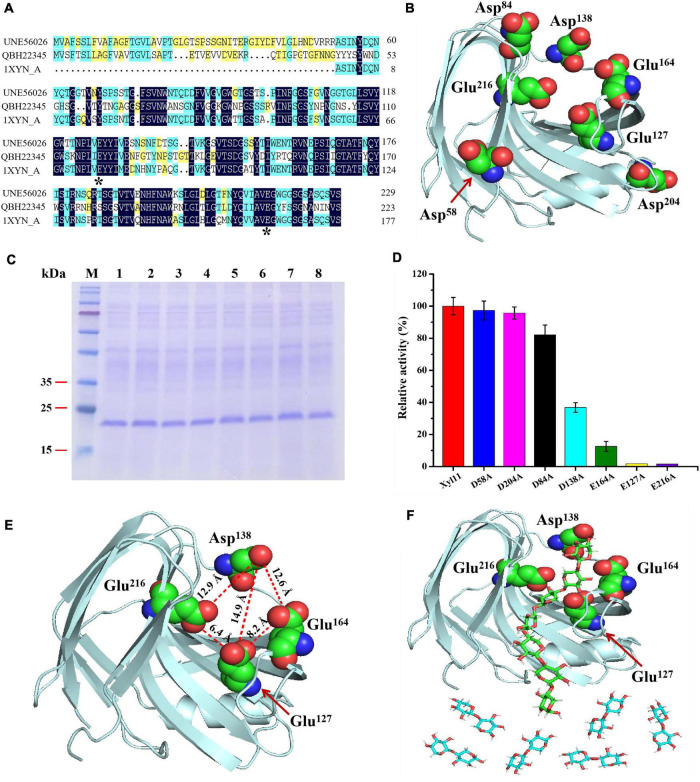
Elucidation of degradation patterns of Xyl11 via site-directed mutagenesis. **(A)** Multiple protein sequence alignment of Xyl11 (UNE56026) with other GH11 xylanases from *T. asperellum* ND-1 (QBH22345) and *T. reesei* (1XYN_A). **(B)** 3D structure simulation of Xyl11 using SWISS-Model software. Potential catalytic residues for mutants were labeled. **(C)** SDS-PAGE analysis of Xyl11 and its mutants. Lanes: M, standard protein markers; 1, Xyl11; 2, D58A; 3, D84A; 4, E127A; 5, D138A; 6, E164A; 7, D204A; 8, E216A. **(D)** Enzyme activities of Xyl11 and its mutants. **(E)** Schematic of Xyl11 active center, displaying distances (Å) among catalytic residues. **(F)** Molecular docking studies of Xyl11 showing substrate xylan in the confirmed active sites.

Based on multiple sequence alignments with other GH11 β-xylanase amino acid sequences, Glu^127^ and Glu^216^ are displayed in highly conserved domains of the catalytic site in Xyl11 (Figure 4A). Analysis of the 3D structure of Xyl11 by molecular dynamics simulation was performed (see text footnote 4). Similar to that of GH11 endo-1,4-xylanase XYNI from *T. reesei* (PDB ID: 1xyn.1), the protein structure of Xyl11 possessed a conservative β-jelly-roll structure (Figure 4B), which is twisted, forming a cleft where the catalytic site is located (Törrönen and Rouvinen, 1995). Moreover, several amino acid residues (especially Glu^127^ and Glu^216^), Asp^58^, Asp^84^, Asp^138^, Asp^204^, and Glu^164^ are situated in the core of catalytic cavity according to the simulated 3D structure of Xyl11 (Figure 4B). It has been reported that amino acids located in the active site of glycoside hydrolases possibly have an important role in substrate binding, catalysis and product release (Nakamura et al., 2013).

All of the potential catalytic residues (Figure 4B) were mutated to alanine residues with short side chains by site-directed mutagenesis. Mutant xylanases D58A, D84A, D138A, D204A, E127A, E164A, and E216A migrated as single protein bands on SDS-PAGE with a size of ∼22 kDa, as with the control (Figure 4C). There was no differences in enzyme activity between D58A, D84A, D204A, and normal xylanase Xyl11 (Figure 4D). However, mutations of D138A and E164A resulted in a drastic decrease (∼ 63 and 87%, respectively) in Xyl11 enzyme activity (Figure 4D), indicating the auxiliary function of Asp^138^ and Glu^164^ toward xylan degradation. Furthermore, no enzyme activity was detected for mutants E127A and E216A (Figure 4D), suggesting that Glu^127^ and Glu^216^ were the critical sites for Xyl11 activity. Ko et al. (1992) proposed that Glu^93^ and Glu^182^ were the best candidates for the crucial catalytic sites of xylanase from *Bacillus pumilus*, whereas for a GH11 xylanase (TlXynA) from *Thermomyces lanuginosus*, two aromatic residues, Tyr^96^ and Tyr^180^, were identified as necessary for catalysis and their mutants significantly reduced enzymatic activities, by almost 95% (Wu et al., 2020).

The hydrolysis patterns of β-xylanases from various GHs families, and even those belonging to the same GHs family but from different microorganisms, vary (Liu et al., 2017). As mentioned above, X3 was converted to high quantities of X2 and a small amount of X1 (Figure 3D), suggesting that Xyl11 possessed transglycosylation activity as well as hydrolysis capacity. To better understand the degradation pattern of Xyl11, molecular docking analysis was performed by using the X3 as the ligand, since X3 was the XOS with minimum Mw that could be efficiently degraded by Xyl11. Interestingly, the mutant site D138A is on the opposite side of the enzyme’s active region (Glu^127^ and Glu^216^) (Figure 4E). Therefore, we reasoned that Asp^138^ could affect the catalytic cavity of the enzyme indirectly, and thus affect its catalytic activity. Furthermore, the Asp^138^-Glu^216^, Asp^138^-Glu^127^, and Asp^138^-Glu^164^ inter-residue distances are ∼12.9, 14.9, and 12.6 Å, respectively (Figure 4E), providing enough space for placement of X2/X3 (between Asp^138^-Glu^216^ and Asp^138^-Glu^127^) and of X3/X4 (between Asp^138^-Glu^216^ and Asp^138^-Glu^164^), as the lengths of X1, X2, X3, and X4 are about 5.5, 10.3, 14.8, and 19.7 Å, respectively. Based on mutants activity and 3D structure analyses, Asp^138^ can constrain X3 tightly to the active center, whereas the catalytic sites (Glu^216^ and Glu^127^) are responsible for hydrolysis activity, and Glu^216^ and Glu^164^ are essential sites for transglycosylation action. This specific structural property of Xyl11 might be the reason for the high proportion of X2 in the degradation products of xylan (DP ≥ 3) (Figure 4F). These findings expand the functional diversity of fungal β-xylanases and offer significant reference information to guide the rational design of other GH families.

### Degradation of beechwood xylan by synergistic action of Xyl11 and commercial β-xylosidase

Recently, xylan-hydrolyzing enzymes have got a lot of attention owing to their potential utilization in developing eco-friendly technologies (Knob and Carmona, 2010; Lagaert et al., 2014; Golgeri et al., 2022). The synergism of β-xylanases and β-xylosidases is important for hydrolyzing xylans efficiently and completely, because β-xylosidases can further hydrolyze X2 and XOSs into X1 and alleviate the inhibitory effect on xylanases by their degradation products (Carvalho et al., 2018). As the final hydrolysate of xylan, X1 can be served as not only a general material in producing Maillard flavorings (Zhao et al., 2019) but also a precursor to prepare high-value products, such as xylitol and succinic acid (Rahmani et al., 2019; Söderling and Pienihäkkinen, 2020).

**FIGURE 5:**
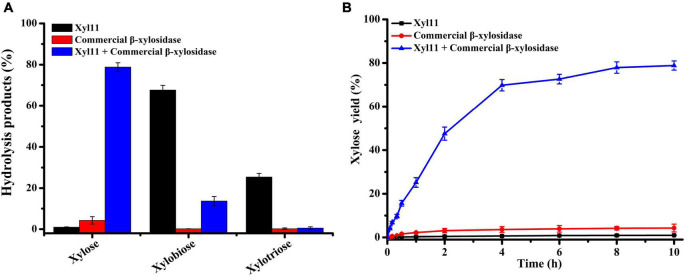
HPLC analysis of degradation products of beechwood xylan. **(A)** Hydrolysis of beechwood xylan by Xyl11 and commercial β-xylosidase individually or in combination. **(B)** Time course profile of xylose production from beechwood xylan.

The capability of Xyl11 and commercial β-xylosidase individually or in combination to degrade beechwood xylan was investigated under simulated gastric conditions (pH 4.0). The yield of X1 and XOSs from the hydrolysis products using different enzymatic degradation methods is shown in Figure 5. Xyl11 alone could produce a large amount of XOSs (67.58% X2 and 25.38% X3) and a trace amount of X1 via hydrolytic activity and transglycosylation (Figure 5A). Conversely, commercial β-xylosidase could hardly degrade beechwood xylan alone, and the yield of X1 was very low, only 4.28% (Figure 5A). On the other hand, in the combined reactions, the amount of X2 and X3 remarkably decreased compared to the degradation products obtained using Xyl11 individually (Figure 5A). Compared with the major degradation product (4.28% X1 at 10 h) obtained using commercial β-xylosidase alone, employing Xyl11 together with it leaded to the X1 yield from enzymatic hydrolysis of beechwood xylan was dramatically increased by 78.84% (Figure 5B), which was comparable with that of *Dictyoglomus thermophilum* β-xylosidase and *Aspergillus niger* NL-1 xylanase in the sequential hydrolysis reaction (xylose yield of 89.9%) (Li et al., 2021); this indicated synergism effects between Xyl11 and commercial β-xylosidase during the degradation process, in which xylan was converted into XOSs (X2 and X3) by the action of Xyl11 and further effectively cleaved into X1 by commercial β-xylosidase. In addition, it is noteworthy that the synergy degree of beechwood xylan by Xyl11 and commercial β-xylosidase degradation was 15.02, which was slightly higher than or comparable with other reported values (Cintra et al., 2017; Liu et al., 2019). The synergistic action of Xyl11 and commercial β-xylosidase could remarkably reduce energy and costs required in terms of improving X1 production, which was a sustainable and environmentally friendly way to produce value-added biomolecules from agricultural waste. Xyl11 is therefore a potentially cost-effective enzyme for industrial applications, e.g., in feedstuff and biofuel production.

## Conclusion

A novel acidic β-xylanase (Xyl11) from *T. asperellum* ND-1 with high hydrolytic activity toward beechwood xylan was identified and characterized. This is the first report showing that codon optimization of the *T. asperellum* acidic β-xylanase gene in *P. pastoris* effectively enhanced the activity of Xyl11 (two-fold). Xyl11 has an optimum pH of 3.0 and displays excellent pH stability under acidic conditions (pH 2.0–5.0). It could rapidly hydrolyze xylan to yield mainly X2 almost without X1 via hydrolysis activity as well as transglycosylation function. Site-directed mutagenesis analysis suggested that the Xyl11 residues, Glu^127^, Glu^164^, and Glu^216^, are the essential catalytic sites, with Asp^138^ possessing an auxiliary function. More importantly, enzyme synergistic effect of Xyl11 and commercial β-xylosidase could remarkably reduce energy and costs required in terms of improving xylose production (by 78.84%), which is of great significance for the biosynthesis of high-value chemicals/industrial products, e.g., biofuels and renewable materials.

## Data availability statement

The datasets presented in this study can be found in online repositories. The names of the repository/repositories and accession number(s) can be found in the article/Supplementary material.

## Author contributions

FZ conceived and designed the study and supervised the present study. FZ and HZ carried out the experiments of enzymatic properties. FZ, JC, JZ, and WC collected the samples and analyzed the results. FZ and AB prepared the manuscript. All authors have read and agreed to the published version of the manuscript.

## Footnotes

1
https://services.healthtech.dtu.dk/service.php?SignalP-5.0

2
http://www.jcat.de/

3
https://blast.ncbi.nlm.nih.gov/Blast.cgi

4
https://swissmodel.expasy.org

5
http://www.cazy.org/
