# Supplementary material for: Biochemical characterization of a novel acidophilic β-xylanase from Trichoderma asperellum ND-1 and its synergistic hydrolysis of beechwood xylan
Source: Front Microbiol. 2022 Sep 15;13:998160. doi: 10.3389/fmicb.2022.998160 (PMC9527580; doi:10.3389/fmicb.2022.998160)
Supplement: Supplementary file 1 [file Data_Sheet_1.pdf]

Supplementary Material

Supplementary Table 1. Oligonucleotide primers used in this study.

Primers	Sequence (5´- 3´)
Xyl11-F	GGTTCTGCATCTCAGAGTGTTAGC
Xyl11-R	TTAATTGCTA ACACTC
AOX-F	GACTGGTTCCAATTGACAAGC
AOX-R	GCAAATGGCATTCTGACATCC
***Xyl11* gene copy number**	
pUC-F	AGCAGATTACGCGCAGAAAAAAAG
xyl11 internal reverse primer-R	GCTAACACTC TGAGATGCAG AACC
oxyl11 internal reverse primer-R	ACTGATTGACTAGCACTTCCAG
**Xyl11 mutants**	
D58A-F	TACGCTCAAAACTACCAAACCGGTGGTACTGTTAACTACTC
D58A-R	GGTAGTTTTGAGCGTAATTAATAGAAGCTCTTCTTCTAACATCGTTATGCAAACC
D84A-F	CTGGAATACTCAAGCTGATTTTGTTGTTGGAGTGGGTTGGGGTACCGG
D84A-R	ATCAGCTTGAGTATTCCAGTTAACGGAAAAACCAGTGGAGGATGG
E127A-F	CCATTGGTTGCGTATTATATCGTTGAATCTAACTCTAACTTCGATACTTCCGG
E127A-R	TAATACGCAACCAATGGATTAGTAGTCCAACCATAAACAGAC
D138A-F	TCGCTACTTCCGGTACTGTTAAGGGTTCTGTTACCAGTG
D138A-R	CAGTACCGGAAGTAGCGAAGTTAGAGTTAGATTCAACGATATAATACTCAACCA ATGG
E164A-F	AACGCACCTTCTATTCAAGGTACTGCTACTTTTAACCAATACATTTCTATCAG
E164A-R	CCTTGAATAGAAGGTGCGTTAACTCTGGTATTTTCCCAAATAGTATAAGAAGAT CCATCACTGG
D204A-F	GGGTTTGGCTCTTGGTACTTTTAACTATCAAGTTATCGCTGTTGAAGGTTGG
D204A-R	GTACCAAGAGCCAAACCCAAGGACTTCCAAGCGTTGAAGTGGTTTTCAACAGTA ACGGTTCC
E216A-F	TATCGCTGTTGCAGGTTGGGGAGGATCTGGAAGTGCTAGTCAATCAGTTTC
E216A-R	CCAACCTGCAACAGCGATAACTTGATAGTTAAAAGTACCAAGATCCAAACCCAA GGAC

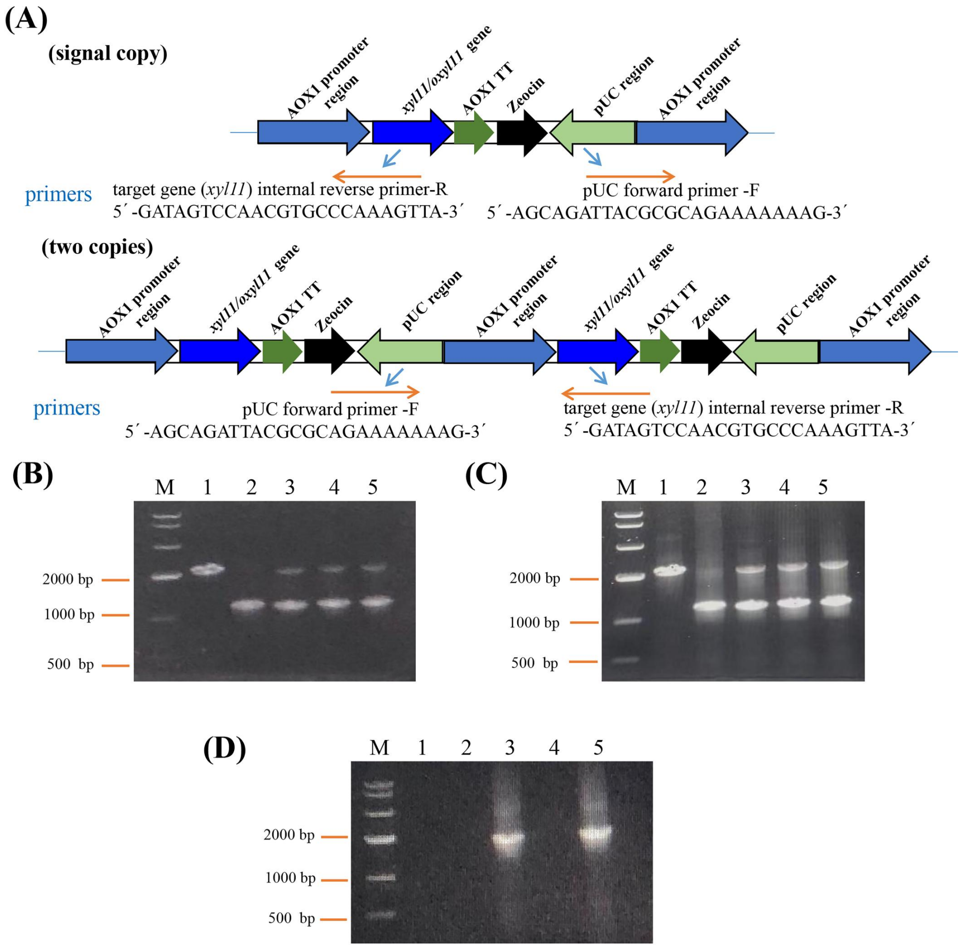

Supplementary Figure 1. Confirmation of recombinant strains by PCR. (**A**) Schematic diagram of single exchange homologous recombination of *P. pastoris*. Identification of engineered strains α-Xyl11 (**B**) and α-oXyl11 (**C**) using PCR primer AOX-F/ AOX-R. Lanes: M, DNA markers; 1, control strain X-33; 2, recombinant plasmids pPICZα-*xyl11* and pPICZα-*oxyl11*; 3-5, recombinant strain α-Xyl11 and α-oXyl11. (**D**) Confirmation of *xyl11* gene copy number in constructed *P. pastoris* strains by PCR with primer pair (pUC-F/ target gene (*xyl11* or *oxyl11*) internal reverse prime). Lanes: M, DNA markers; 1, control strain X-33; 2, recombinant strain α-Xyl11; 3, recombinant plasmid pPICZα-*xyl11*; 4, recombinant strain α-oXyl11; 5, recombinant plasmid pPICZα-*oxyl11*.

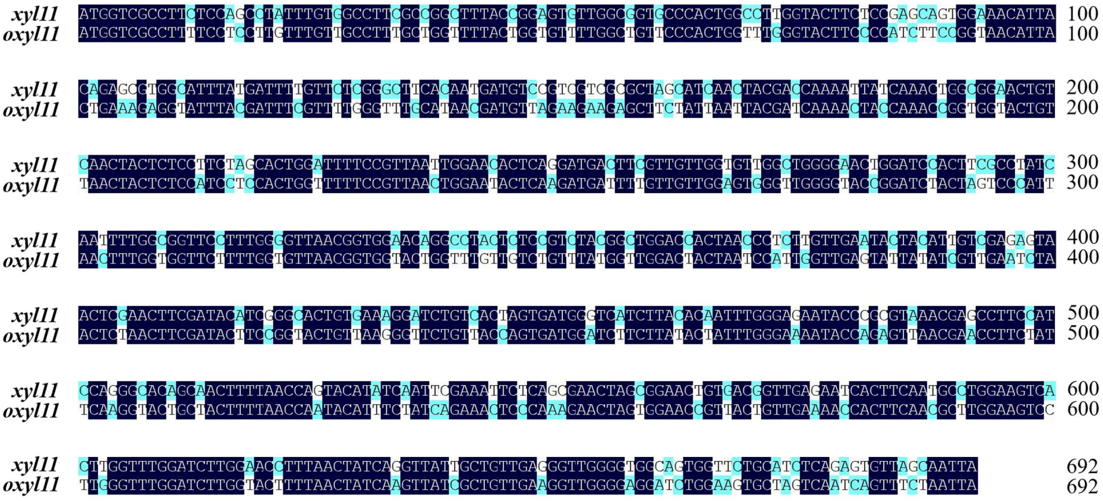

Supplementary Figure 2. Alignment of original β-xylanase gene (*xyl11*) sequence and optimized type gene (*oxyl11*) sequence.
